# Prognostic factors of head and neck cutaneous squamous cell carcinoma: a systematic review

**DOI:** 10.1186/s40463-021-00529-7

**Published:** 2021-09-07

**Authors:** Joshua Lubov, Mathilde Labbé, Krystelle Sioufi, Grégoire B. Morand, Michael P. Hier, Manish Khanna, Khalil Sultanem, Alex M. Mlynarek

**Affiliations:** 1 Department of Otolaryngology Head and Neck Surgery, Sir Mortimer B. Davis-Jewish General HospitalMcGill University, 3755 Côte Ste-Catherine Road, Montreal, QC H3T 1E2 Canada; 2 Department of Dermatology, Sir Mortimer B. Davis-Jewish General Hospital, McGill University, Montreal, QC Canada; 3 Department of Radiation Oncology, Sir Mortimer B. Davis-Jewish General Hospital, McGill University, Montreal, QC Canada

**Keywords:** Carcinoma, Squamous cell, Sentinel lymph node biopsy, Mohs surgery, Skin neoplasms, Squamous cell carcinoma of head and neck

## Abstract

**Background:**

Head and neck cutaneous squamous cell carcinoma (HNCSCC) is a non-melanoma skin cancer that is mostly caused by solar ultraviolet radiation exposure. While it usually has an excellent prognosis, a subset of patients (5%) develops nodal metastasis and has poor outcomes. The aim of this study was to systematically review the literature and evaluate the prognostic factors of HNCSCC in order to better understand which patients are the most likely to develop metastatic disease.

**Methods:**

A comprehensive literature search was performed on PubMed and EMBASE to identify the studies that evaluated the prognostic factors of HNCSCC. Prognostic factors were deemed significant if they had a reported *p*-value of < 0.05. Proportions of studies that reported a given factor to be statistically significant were calculated for each prognostic factor.

**Results:**

The search yielded a total of 958 citations. Forty studies, involving a total of 8535 patients, were included in the final analysis. The pre-operative/clinical prognostic factors with the highest proportion of significance were state of immunosuppression (73.3%) and age (53.3%); while post-operative/pathological prognostic factors of importance were number of lymph nodes involved with carcinoma (70.0%), margins involved with carcinoma (66.7%), and tumor depth (50.0%).

**Conclusion:**

This systematic review is aimed to aid physicians in assessing the prognosis of HNCSCC and identifying the subsets of patients that are most susceptible to metastasis. It also suggests that immunosuppressed patients with a high-risk feature on biopsy, such as invasion beyond subcutaneous fat, could possibly benefit from a sentinel lymph node biopsy.

**Graphical abstract:**

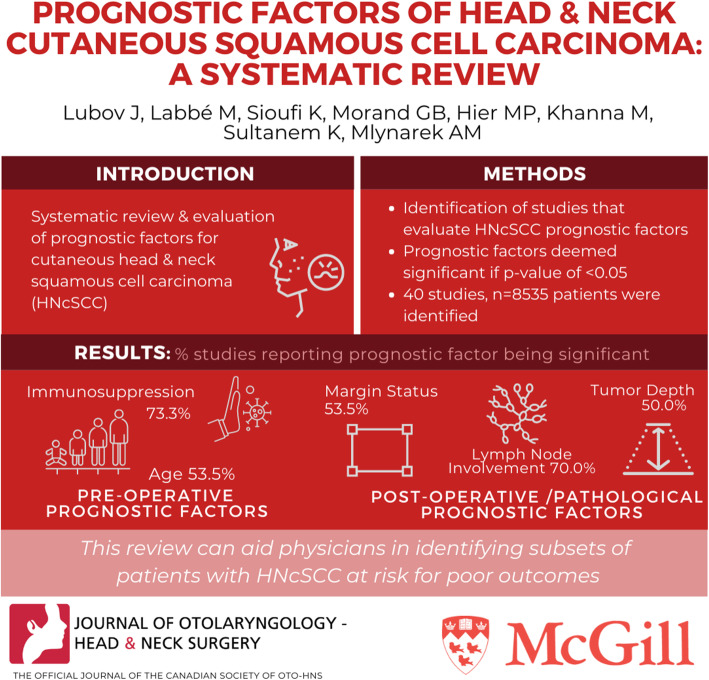

**Supplementary Information:**

The online version contains supplementary material available at 10.1186/s40463-021-00529-7.

## Background

Cutaneous squamous cell carcinoma (cSCC) is the second most common non-melanoma skin cancer (NMSC) [1]. As sun exposure is a major risk factor for cSCC, they arise commonly from the head and neck, commonly the ear, cheek, lip, and scalp [2, 3].

The recent literature reports a sharp increase in incidence of cSCC [4–6]. Over the last 30 years, longitudinal studies based in Canada and Australia have shown a 50–300% increase in the incidence of primary cSCC [1, 7, 8]. Despite the high incidence rates, it is reported that less than 5% of patients develop nodal metastases [9]. The majority of patients with cSCC undergoes electrodessication and curettage, cryosurgery, or Mohs’ surgery, and have an excellent prognosis. However, there is a subset of patients in which these therapies are unsuccessful and where cSCC appears to be far more aggressive, often resulting in metastasis and recurrence [1]. The poor survival rates for metastatic cSCC highlights the importance of identifying the patients who are susceptible to metastatic disease and poor outcome. Indeed, it has been shown that metastasis in cSCC patients is associated with a 50% decrease in five-year survival probability [10]. To date it remains however ambiguous to what defines this subset of patients. This may be accounted for by the inconsistency of the prognostic factors reported in the literature.

Moreover, in the last decade, sentinel lymph node biopsy (SLNB) has become a common practice in staging melanoma and has been utilized in other invasive cancers, such as oral squamous cell carcinoma [11, 12]. SLNB allow for pathological examination of the first echelon drainage node, helpful for determining accurate prognoses and therapy [11]. As 95% of cSCC patients have an excellent prognosis, it remains unclear as to which patients should be offered a SLNB.

Due to this therapeutic dilemma, and the fact that no study has evaluated in a thorough manner yet the prognostic factors associated with metastasis in head and neck cutaneous squamous cell carcinoma (HNCSCC), we felt appropriate to conduct a systematic review. The aim of the study was to determe the most significant negative prognostic factors in HNCSCC. This would provide an insight into which patients are more likely to develop metastasis and which could possibly benefit from a sentinel lymph node biopsy.

## Materials and methods

### Search strategy

We extracted information from all eligible publications using a standardised data extraction sheet and report the review according to equator network PRISMA guidelines. We searched for studies in the electronic databases PubMed and EMBASE for studies that analyzed prognostic factors in patients with HNCSCC. The following key words, as well as their synonyms, and abbreviations, were employed in the search strategy: Squamous cell carcinoma, Head and neck, Prognosis, and Cutaneous (Additional file 1). In order not to miss any appropriate study, we did not apply any time in our search. Only article published in English were considered. The reference lists of review articles were screened for potentially eligible studies.

The selection of studies involved an initial screening of the title and the abstract. In doubtful cases we obtained the full text. We entered articles in a data management software and eliminated the duplicates (Endnote 8®, Thompson Reuters Inc.)

### Inclusion and exclusion criteria

Titles and abstracts were screened in order to identify the relevant studies. Articles were included based on the following criteria: (1) related to cutaneous squamous cell carcinoma; (2) related to cancers of the head and neck region; (3) Studies that were investigating prognostic factors. The full text of any papers missing abstracts or that had ambiguous titles and abstracts were screened in order to determine if they were relevant. After this elimination process, the following exclusion criteria were used to determine which articles were eligible for the data extraction: (1) No data extraction possible for head and neck only; (2) Studies that did not include prognostic factors as part of statistical analysis; (3) Studies that failed to perform multivariate analysis to assess significance of prognostic factors; (4) Literature reviews, case reports were also excluded.

### Data extraction

The selection and data process were individually done by three authors (JL, ML, KS). The initial selection was performed before retrieval of full manuscript based on the title and in the abstract. Manuscripts were fully reviewed if they are within the inclusion and exclusion criteria. The last author (AMM) was consulted in case of disagreement. All the data that was retrieved from the studies that qualified for the final inclusion was compiled into spreadsheets (Tables 1 and 2). This data included the author, population, sample size, anatomical location of the primary HNCSCC, primary outcome evaluated in the study and the significance of the following prognostic factors: age, sex, parotid staging, neck staging, tumor size, tumor thickness, differentiation grade, anatomical site, perineural invasion, lymphovascular invasion, extranodal extension, margins involved with carcinoma, recurrence, state of immunosuppression and the number of lymph nodes involved with carcinoma. Any other significant prognostic factors included in the studies were noted as well. Several studies tackled adjuvant radiotherapy as a prognostic factor. However, this treatment modality is usually offered and given depending on the presence of other prognostic factors, which is why it is not discussed in the following literature review.

**Table 1:** Study demographics, sample size, and primary anatomic locations

Author	Population	Sample Size	Primary Anatomic Location
Andruchow et al. 2006 [13]	Australia	322	Head & Neck
Audet et al. 2004 [14]	Canada	56	Head & Neck
Brantsch et al. 2008 [5]	Germany	615	Lip (26%), ear (13%), other (61%)
Bobin et al. [15]	France	35	Ear (12%), temple (12%), scalp (6%), recurrent lesion (6%)
Ch'ng et al. 2008 [16]	New Zealand	170	External ear (26.5%), cheek (12.9%), temporofrontal (18.8%), scalp (11.2%), lower lip (10.6%), upper lip (0.59%), nose (3.53%), eyelid (1.18%), neck (2.35%), multiple head and neck (12.4%)
Chua et al. 2002 [17]	Australia	52	Head & Neck
Clark et al. 2012 [9]	Australia	603	Head & Neck
Creighton et al. 2018 [18]	US	62	Head & Neck
de Koning et al. 2009 [19]	Netherlands	99	Oral cavity and oropharynx
Ebrahimi et al. 2013 [20]	Australia	229	Face (55.4%), external ear (20.1%), scalp (17.1%), neck (5.7%), other H&N (1.7%)
Eigentler et al. 2017 [21]	Germany	1434	Ear (14.4%), Lip (lower vermilion surface) (6.4%), Other (79.2%), Face, other (65.6%), Body, other (13.7%)
Forest et al. 2010 [22]	Australia	215	Head & Neck
Garcia-Pedrero et al. [23]	Chile	100	Head & Neck
Goh et al. 2012 [24]	Australia	66	Head & Neck
Haisma et al. 2016 [25]	Netherlands	336	Anterior aspect of the scalp (19.3%), posterior aspect of the scalp (10.8%), neck (4.25), nose (10.5%), lip (16.7%), ear (22.2%), other (16.3%)
Harris et al. 2017 [26]	US	212	Ear (22.65), cheek/temple (28.3%), lip (10.4%), neck (2.4%), nose (9.4%), periorbital (5.7%), scalp (15.6%)
Hinerman et al. 2008 [27]	US	117	Head & Neck
Hirshoren et al. 2017 [28]	Australia	149	Scalp (35%), preauricular (5%), ears (21%), nose (21%), lip lower/upper (11%), neck (1.8%), cheeks (9%), eye lids (0.9%), postauricular (95), chest (0.9%)
Jambusaria-Pahlajani et al. 2013 [29]	US	237	Lip or ear (58%), other (42%)
Kelder et al. 2012 [30]	Australia	164	Head & Neck
Khandelwal et al. 2016 [31]	US	37	Facial
Khurana et al. 1995 [32]	Australia	75	Head & Neck
Kreppel et al. 2013 [33]	Germany	63	Lower lip (34.9%), upper lip (1.6%), preauricular region (12.7%), nose (12.7%), ear (11.1%), front (4.8%), neurocranium (3.2%), cheek (19.0%)
Kyrgidis et al. 2010 [34]	Greece	315	Forehead and temple (17.5%), eyelids and periocular skin (13.0%), auricle and periauricular skin (23.8%), cheek (32.7%), nasal area (8.89%), neck (4.13%)
Makki et al. 2013 [35]	Canada	54	Head & Neck
McLean et al. 2013 [36]	Australia	100	Head & Neck
Mizrachi et al. 2013 [37]	Israel	71	Auricle (19.7%), cheek (16.9%), scalp (11.3%), preauricular region (9.86%), forehead (8.45%), temple (5.63%), chin (4.23%), eyelid (4.23%), neck (4.23%), lip (4.23%), nose (2.82%), other (8.42%)
Moore et al. 2005 [38]	US	193	Periauricular (30.6%), forehead/temple (20.2%), cheek (14.0%), nose (9.85%), scalp (8.29%), neck (6.22%), lower lip (4.66%), periorbital (5.2%), upper lip (2.07%)
O’Brien et al., 2002 [3]	Australia	87	Head & Neck
Oddone et al. 2009 [39]	Australia	250	Head & Neck
Palme et al. 2003 [40]	Australia	126	Head & Neck
Peat et al. 2012 [41]	New Zealand	170	Head & Neck
Pramana et al. 2012 [42]	Australia	75	Scalp (18.7%), ear (16.0%), face (8.0%), neck (2.67%), unknown (54.7%)
Schmidt et al. 2015 [43]	Australia	113	Head & Neck
Dyall-Smith et al. 2016 [45]	Australia	442	Head & Neck
Sweeny et al. 2014 [44]	US	238	Cheek (44%), orbit (2%), forehead (2%), preauricular (115), pinna (20%), postauricular (7%), temporal (6%), unknown (65)
Vasan et al. 2018 [46]	Australia	326	Skin of face (19%), external ear (12%), skin of nose (17%), skin of scalp and neck (10%), skin of lip (35), eyelid (< 1%), unknown (28%)
Veness et al. 2005 [6]	Australia	167	Head & Neck
Tseros et al. 2016 [47]	Australia	238	Head & Neck
Wang et al. 2012 [48]	Australia	122	Lip (27%), posterior scalp (9%), cheek (9%), nose (9%), ear (9%), other (37%)

**Table 2:**
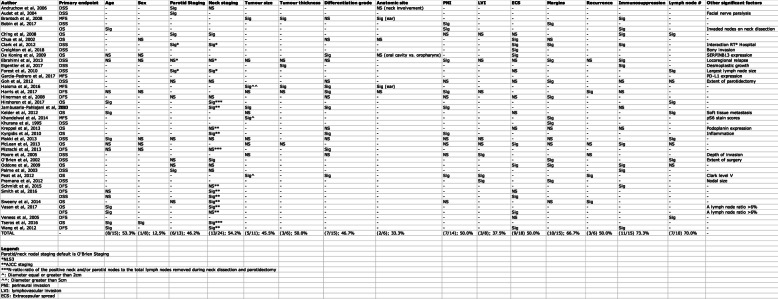
Significance of prognostic factors in head and neck cutaneous squamous cell carcinoma

### Data analysis

The prognostic factors evaluated in each study were classified as either significant or not significant. Significance was defined as having a *p*-value of < 0.05 and a 95% confidence interval. Prognostic factors’ significance calculated with univariate analyses were disregarded. The total amount of times that each prognostic factor was found significant and non-significant in the literature was recorded and proportions were calculated.

## Results

**Fig. 1:**
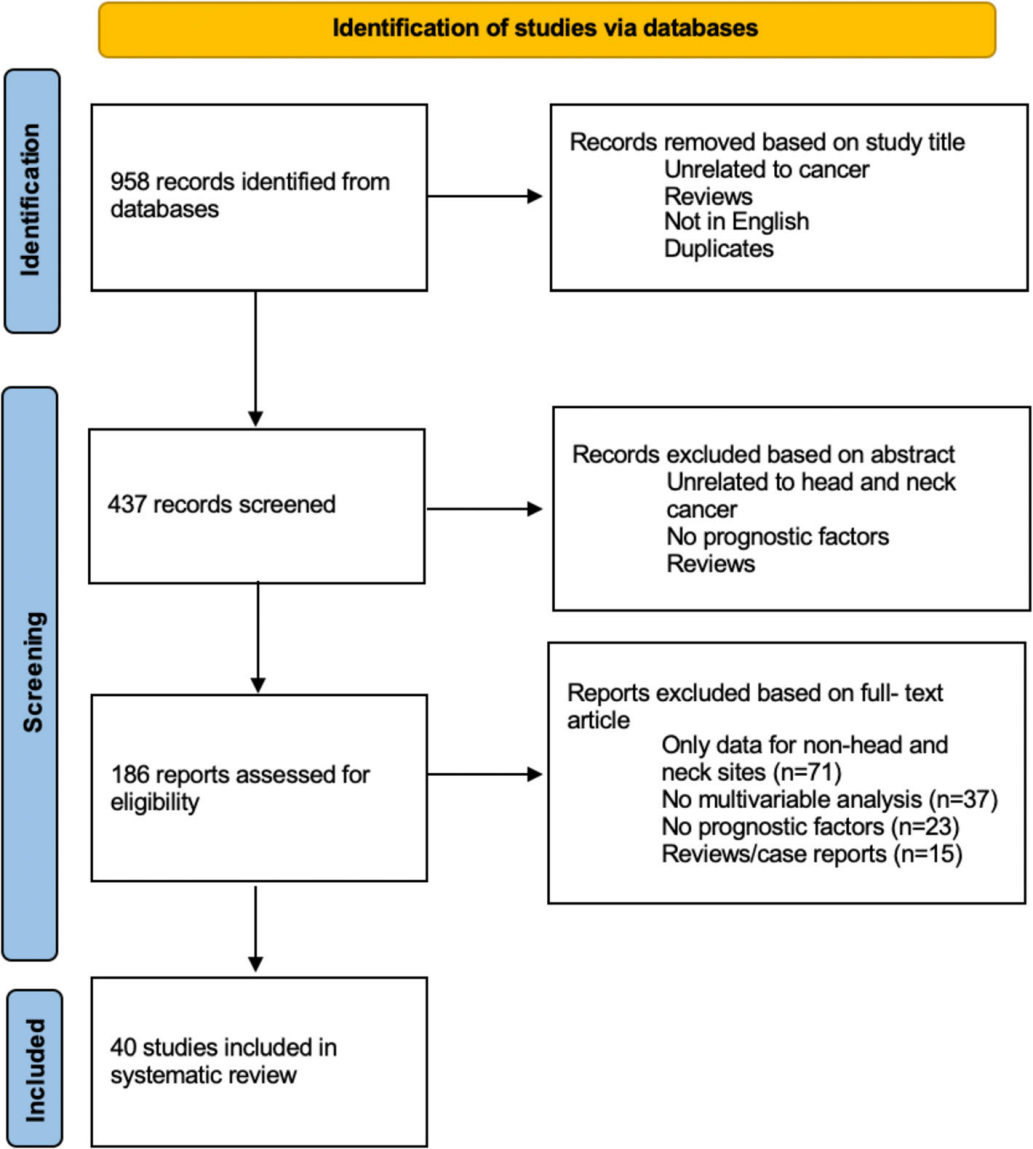
Study selection flow chart

Our search strategy yielded a total of 958 articles after excluding duplications. Following the review of titles and abstracts, 175 full-text articles were reviewed. In total, 40 [3, 5, 6, 9, 13–20, 22, 24–43, 45–48] papers were found to meet the complete list of inclusion and exclusion criteria as defined in the methods and were included in our review (Fig. 1).

The total number of patients included in this systematic review was 8535 out of 40 studies, with the smallest study including 35 patients, while the biggest included 1434 patients. All patients had, according to the inclusion criteria, primary lesion located in the head or neck region. Out of the 40 studies included, 20 (50.0%) were conducted in Australia. The other populations included were the United States (17.5%), Germany (7.5%), Canada (5.0%), New Zealand (5.0%), Netherlands (5.0%), Israel (2.5%), Greece (2.5%), France (2.5%), and Chile (2.5%) (Table 1).

The analysis of patient factors and features found pre-operatively or on biopsy lead to the following results (Table 2): Out of the fifteen studies that assessed age, eight (53.3%) of them stated that age was a significant prognostic factor. Out of the eight that assessed sex, one deemed it as a significant factor. Only 2 studies out of six (33.3%) that assessed for the involved anatomical site found that it was significant. Similarly, three of six (50.0%) studies indicated that having a recurrence was significant. Out of the 15 studies that assessed immunosuppression, eleven (73.3%) suggested that it was significant. Tumor depth was found to be significant in three of six (50.0%) studies. Seven out of the 15 (46.5%) studies that assessed the histological differentiation grade claimed that it was a significant prognostic factor.

**Fig. 2:**
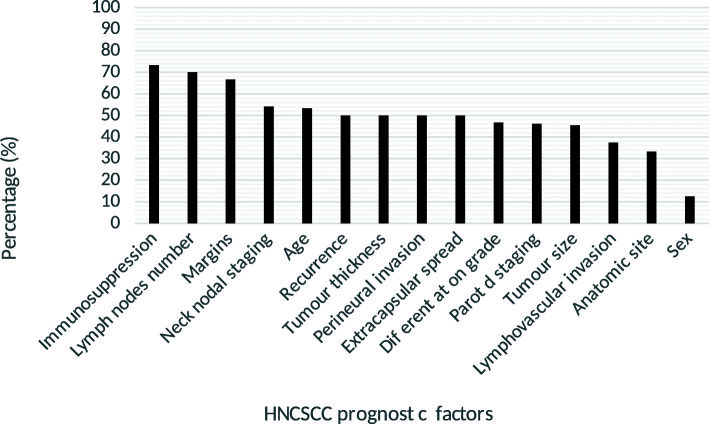
Percentage of significance of prognostic factors

The analysis of the factors known post-operatively lead to the following results. Five of eleven (45.5%) studies found that tumor size was a significant prognostic factor. Nine of 18 (50.0%) studies indicated that extranodal extension was significant. Ten of 15 (66.7%) studies stated that margins involved with carcinoma was a significant prognostic factor. For the parotid and neck staging, six out of 13 (46.2%) studies suggested that parotid staging was a significant factor and thirteen out of the 24 studies (54.2%) found that neck staging was significant. Out of the ten studies that considered the number of lymph nodes (multiple nodes, ≥2) affected by carcinoma, 7 (70.0%) indicated that it was a significant prognostic factor. Seven of fourteen (50.0%) and three of eight (37.5%) studies suggested that perineural invasion and lymphovascular invasion, respectively, were significant prognostic factors. The ranking of the prognostic factors is shown in Fig. 2.

The primary outcomes of the included studies varied with 39.5% (17/43) of studies reporting disease-specific survival, 32.6% (14/43) reporting overall survival, 18.6% (8/43) reporting disease-free survival, and 9.3% (4/43) reporting metastasis free survival.

## Discussion

To our knowledge, we present the first systematic review looking at prognostic factors for HNCSCC. Given the low rate of metastasis from HNCSCC lesions, it can be challenging to identify the patients who are at high risk of having metastatic disease. We believe this review could help identify patients that would require a closer follow-up and those that could possibly profit from a SLNB.

### State of immunosuppression

Out of the prognostic factors evaluated in the present study, immunosuppression was the most consistently reported significant prognostic factor (73.3%). It is postulated that a state of immunosuppression increases the risk of metastasis, as well as it decreases overall survival [13, 16, 17, 19, 20]. In their multivariate analysis, Brantsch et al. [5] reported that metastasis-free survival time was considerably affected in the immunosuppressed patients (HR 4.32; *p* = 0·0035). Oddone et al. [39] also deemed immunosuppression as a powerful prognostic indicator (HR 3.13; *p* = 0·006) and included it in their ITEM (immunosuppression, treatment, extranodal extension, and margin status) score, which suggests high-risk features for primary HNCSCC.

Solid-organ transplant recipients make up the majority of the immunosuppressed population. It was found that cSCC is the most common malignancy for patients who have received renal transplants, and these have an up to 82-fold increased likelihood of developing invasive cSCC compared to the general population [49]. As solid-organ transplant procedures are becoming more common [6], immunosuppression must be considered when evaluating risk stratification and prognosis of HNCSCC patients. Future studies shall allow better risk stratification according to the type and degree of immunosuppression,

### Tumor factors

Tumor thickness (especially above 6 mm) and/or infiltration into the subcutaneous fat was found consistently to be a significant negative prognostic factor in HNCSCC. Depth of invasion has been a major prognostic factor for melanoma (Breslow thickness) and also recently been integrated in the stating system for oral squamous cell carcinoma. Further, perineural invasion (PNI) and lymphovascular invasion (LVI) were found to have a significant prognostic value in 50 and 37.5% of the included studies, respectively. Histological differentiation was also significant in 46.7% of studies, with all authors agreeing that a poorer grade of cellular differentiation lead to worse outcomes for patients.

### Lymph node involvement and discrepancies in parotid and neck staging system

The number of lymph nodes affected by carcinoma showed to be a significant prognostic factor in 70.0% of studies. The higher number of lymph nodes (≥ two nodes) involved with HNCSCC, the worse the prognosis [3, 22, 30]. .Forest et al. demonstrated the significance of having multiple lymph nodes involved versus a single one (HR 3.8; *p* = 0·002) [22]. Another retrospective chart review showed that the five-year disease-free survival was significantly better in patients with a single node involved, compared to multiple nodes (75% vs. 64%; *p* = 0.04) [6]. Those findings suggest that patients with more than two lymph nodes should benefit from a closer follow-up.

Moreover, positive parotid and neck disease were found to be significant in 54.2 and 46.2% of the time, respectively. One important finding of our systematic review is the discrepancies in staging HNCSCC in the literature. In fact, there appears to be no standard staging system for this disease. Out of the 22 studies that assessed neck nodal staging, 9 studies used the O’Brien staging system (40.9%) [3], 9 studies used the AJCC staging system (40.9%), and 3 studies used the N1S3 staging system (13,6%) [22]. A study comparing the 7th edition of the AJCC staging system for HNCSCC and the N1S3 staging system stated the N1S3 staging system is superior to the AJCC staging system due to the fact that the N1S3 has statistically better distribution and stratification [9]. Discrepancies in the staging systems used in the literature can account for the inconsistency of the significance of parotid and neck staging. More studies are needed to compare those methods and to define a standard staging system for the field.

### Margin status

The third most consistently significant prognostic factor was margin status. Out of the 15 studies that assessed margin status, 66.7% reported that margins involved with carcinoma lead to a poorer prognosis. A study by Hinerman et al. [27] concluded that positive surgical margins conferred a worse prognosis compared to negative and close [≤5 mm] margins when considering disease-free survival. Interestingly, O’Brien et al. [3] found that patients with advanced disease were more likely to have positive margins. Moreover, O’Brien’s study showed that patients who underwent more radical surgeries were the ones with a higher chance of having positive margins. This can be explained by the fact that large facial primaries are more difficult to excise due to their size, depth and vital surrounding structures. Therefore, involved margins constitute an important prognosis factor in HNSCCS that should always be taken into consideration when evaluating prognosis in patients with HNCSCC.

### Age

Age was found to be a significant prognostic factor in 53.3% of studies. Four studies^23, 42–44^ demonstrated that advanced age was significantly associated with a decreased overall survival. It is postulated that with age, the body accumulates somatic mutations and gets increasingly exposed to risk factors that favor the development of different cancers. Vasan et al. [46], in their retrospective analysis including 326 patients with a mean age of 73.3 years, showed that this factor negatively affected the disease-free survival (DFS) (HR 1.02; *p* = 0.02) and overall survival (HR 1.04; *p* < 0.001). Although statistically significant, the magnitude of the negative effect on DFS is low and unlikely to be of clinical significance. Interestingly, one study in the present review demonstrated a contrary observation. A retrospective study^27^ showed that older patients diagnosed with HNCSCC had a better prognosis than younger ones (HR 0.92; *p* = 0.002). Therefore, although age was shown to be a significant factor in approximately half the included studies, the extent of its effect on prognosis and use as a clinically significant marker is questionable. Given the discordance on its significance in the literature, more studies are needed to clarify its prognostic importance in HNCSCC.

### Other findings

Gender was found to be significant in one of all the studies analyzed. Anatomic site and state of recurrence were each only studied in six studies. Extranodal extension (ENE) was significant in 50.0% of studies. Interestingly, ECS, which was included as one of the four prognostic factors on the Oddone’s ITEM score, carries the heaviest prognostic significance amongst them [39].

### Sentinel lymph node biopsy and comparison of results with current literature

Sentinel lymph node biopsy (SLNB) has been widely accepted as a minimally invasive and accurate technique for detecting occult nodal metastases in cutaneous melanoma and breast cancer [12]. However, its use in HNCSCC remains controversial, as it is unclear from the literature whether it has benefits with regards to survival. Ross and Schmults [50] showed in their systematic review that SLNB performed on cSCC in high-risk lesions were positive 21% of the time.

Considering factors related to the primary tumor only, our results suggest that state of immunosuppression and increasing tumor depth, followed by perineural invasion, lymphovascular invasion and poor histological differentiation are important prognostic factors for poor outcomes in HNCSCC. These results are compatible with the literature on cSCC that is not specific to the head and neck region [21, 51]. Based on these results, we created a criterion to help define the subset of head and neck patients that could possibly benefit from a SLNB (Table 3). We believe that a patient post-biopsy with either two major criteria or one major and two minor criteria should be considered as a candidate for SLNB at the time of the surgery. An alternative SLNB would be, if postoperative radiotherapy of the primary tumor is anticipated or very likely, to limit the surgery to resection of the clinical disease without SLNB and treat electively the tumor and the lymphatic drainage with radiotherapy. One possible drawback is the lack of pathological examination of the first echelon node [52]. Prospective studies looking at the benefit and drawbacks of SLNB are definitely needed.

**Table 3:** Major and minor criteria for sentinel lymph node biopsy

Major Criteria	Minor Criteria
• State of immunosuppression • Depth of tumor ≥6 mm or beyond subcutaneous fat	• Perineural invasion • Lymphovascular invasion • Poor histological differentiation

### Limitations

There are several limitations to this systematic review. First, the majority of the included studies were based in Australia, involving 3921 out of the 8535 (45.9%) patients in this review, which may induce a selection bias and could limit the external validity of the study. Another important limitation is the fact that most studies on prognostic factors of cSCC were not specific to the head and neck region. Therefore, despite a large proportion of head and neck population included in most studies (see Table 1), single study results - allowing a meta-analysis - could not be extracted as they were not divided based on anatomical location. We chose to report how often a particular prognostic factor was found to be significant in each study, which gives an idea – although not quantitative – on how consistent a finding is. Lastly, some of our included studies had very small sample sizes, making the potential for beta-error high. In other words, some small study may give this impression that a given clinicopathological factor is not significantly associated with worse prognosis of HNSCC, although this may be due to limited sample size (false-negative). Another limitation of our study is the fact that most prognostic factors reported in the literature did not account for major treatment variables, such as the use of postoperative radiotherapy, for example. Future studies on cSCC should explore those factors, and differentiate results based on anatomical location, in order to expand the data on HNCSCC.

## Conclusion

The prognostic factors for head and neck cutaneous squamous cell carcinoma that were most consistently reported as significant in the literature are a state of immunosuppression, tumor depth, margins involved, number of lymph nodes affected by carcinoma, parotideal disease, and age. This systematic review can aid physicians in assessing the prognosis of patients and possibly identifying the subsets of patients that are most susceptible to metastasis. We believe that immunosuppressed patients with high-risk features could possibly benefit from a sentinel lymph node biopsy at the time of the surgery*.* As some prognostic factors are poorly studied in the literature, more research is needed to assess their value in the prognosis of head and neck cutaneous squamous cell carcinoma.

## Supplementary Information

**Additional file 1.**

## Data Availability

All data generated and analyzed during this study are available in published articles available on the PudMed and EMBASE repositories: https://pubmed.ncbi.nlm.nih.gov, https://www.embase.com/login**.** This material has never been published before and is not currently under consideration by another journal.

## Abbreviations

HNCSCC: Head and neck cutaneous squamous cell carcinoma
cSCC: Cutaneous squamous cell carcinoma
NMSC: Non-melanoma skin cancer
SLNB: Sentinel lymph node biopsy
ITEM: Immunosuppression, treatment, extranodal extension, and margin status
AJCC: American Joint Committee on Cancer
DFS: Disease free survival
ENE: Extranodal extension
PNI: Perineural invasion
LVI: Lymphovascular invasion

## References

[1] Brougham ND, Dennett ER, Cameron R, Tan ST (2012). The incidence of metastasis from cutaneous squamous cell carcinoma and the impact of its risk factors. J Surg Oncol.

[2] O'Hara J, Ferlito A, Takes RP, Rinaldo A, Strojan P, Shaha AR, Rodrigo JP, Paleri V (2011). Cutaneous squamous cell carcinoma of the head and neck metastasizing to the parotid gland--a review of current recommendations. Head Neck.

[3] O'Brien CJ, McNeil EB, McMahon JD, Pathak I, Lauer CS, Jackson MA (2002). Significance of clinical stage, extent of surgery, and pathologic findings in metastatic cutaneous squamous carcinoma of the parotid gland. Head Neck.

[4] Gallagher RP, Hill GB, Bajdik CD, Coldman AJ, Fincham S, McLean DI, Threlfall WJ (1995). Sunlight exposure, pigmentation factors, and risk of nonmelanocytic skin cancer. II. Squamous cell carcinoma. Arch Dermatol.

[5] Brantsch KD, Meisner C, Schonfisch B, Trilling B, Wehner-Caroli J, Rocken M (2008). Analysis of risk factors determining prognosis of cutaneous squamous-cell carcinoma: a prospective study. Lancet Oncol.

[6] Veness MJ, Morgan GJ, Palme CE, Gebski V (2005). Surgery and adjuvant radiotherapy in patients with cutaneous head and neck squamous cell carcinoma metastatic to lymph nodes: combined treatment should be considered best practice. Laryngoscope..

[7] Rogers HW, Weinstock MA, Feldman SR, Coldiron BM (2015). Incidence estimate of nonmelanoma skin Cancer (keratinocyte carcinomas) in the U.S. population, 2012. JAMA Dermatol.

[8] Alam M, Ratner D (2001). Cutaneous squamous-cell carcinoma. N Engl J Med.

[9] Clark JR, Rumcheva P, Veness MJ (2012). Analysis and comparison of the 7th edition American joint committee on Cancer (AJCC) nodal staging system for metastatic cutaneous squamous cell carcinoma of the head and neck. Ann Surg Oncol.

[10] Dwojak S, Emerick KS (2015). Sentinel lymph node biopsy for cutaneous head and neck malignancies. Expert Rev Anticancer Ther.

[11] Silberstein E, Sofrin E, Bogdanov-Berezovsky A, Nash M, Segal N (2015). Lymph node metastasis in cutaneous head and neck squamous cell carcinoma. Dermatol Surg.

[12] Kwon S, Dong ZM, Wu PC (2011). Sentinel lymph node biopsy for high-risk cutaneous squamous cell carcinoma: clinical experience and review of literature. World J Surg Oncol.

[13] Andruchow JL, Veness MJ, Morgan GJ, Gao K, Clifford A, Shannon KF, Poulsen M, Kenny L, Palme CE, Gullane P, Morris C, Mendenhall WM, Patel KN, Shah JP, O'Brien CJ (2006). Implications for clinical staging of metastatic cutaneous squamous carcinoma of the head and neck based on a multicenter study of treatment outcomes. Cancer..

[14] Audet N, Palme CE, Gullane PJ, Gilbert RW, Brown DH, Irish J, Neligan P (2004). Cutaneous metastatic squamous cell carcinoma to the parotid gland: analysis and outcome. Head Neck.

[15] Bobin C, et al. Prognostic factors for parotid metastasis of cutaneous squamous cell carcinoma of the head and neck. Eur Ann Otorhinolaryngol Head Neck Dis. 2018;135(2):99–103. 10.1016/j.anorl.2017.09.006 29100720

[16] Ch'ng S, Maitra A, Allison RS, Chaplin JM, Gregor RT, Lea R, Tan ST (2008). Parotid and cervical nodal status predict prognosis for patients with head and neck metastatic cutaneous squamous cell carcinoma. J Surg Oncol.

[17] Chua MS, Veness MJ, Morgan G, Shakespeare T, Hehir A, Gebski V (2002). Parotid lymph-node metastases from cutaneous squamous-cell carcinomas: treatment outcome and prognostic factors following surgery and adjuvant radiotherapy. Australas Radiol.

[18] Creighton F, et al. Factors affecting survival and locoregional control in head and neck cSCCA with nodal metastasis. Laryngoscope. 2018;128(8):1881–6. 10.1002/lary.27048 29266236

[19] de Koning PJ, Bovenschen N, Leusink FK, Broekhuizen R, Quadir R, van Gemert JT (2009). Downregulation of SERPINB13 expression in head and neck squamous cell carcinomas associates with poor clinical outcome. Int J Cancer.

[20] Ebrahimi A, Clark JR, Ahmadi N, Palme CE, Morgan GJ, Veness MJ (2013). Prognostic significance of disease-free interval in head and neck cutaneous squamous cell carcinoma with nodal metastases. Head Neck.

[21] Eigentler TK, Leiter U, Hafner HM, Garbe C, Rocken M, Breuninger H (2017). Survival of patients with cutaneous squamous cell carcinoma: results of a prospective cohort study. J Investig Dermatol.

[22] Forest VI, Clark JJ, Veness MJ, Milross C (2010). N1S3: a revised staging system for head and neck cutaneous squamous cell carcinoma with lymph node metastases: results of 2 Australian Cancer centers. Cancer..

[23] Garcia-Pedrero JM, et al. J Am Acad Dermatol. 2017. PMID: 28716437. 10.1016/j.jaad.2017.05.047 28716437

[24] Goh RY, Bova R, Fogarty GB (2012). Cutaneous squamous cell carcinoma metastatic to parotid - analysis of prognostic factors and treatment outcome. World Jo Surg Oncol.

[25] Haisma MS, et al. Multivariate analysis of potential risk factors for lymph node metastasis in patients with cutaneous squamous cell carcinoma of the head and neck. J Am Acad Dermatol. 2016;75(4):722–30. 10.1016/j.jaad.2016.06.010 27473455

[26] Harris BN, et al. Factors associated with recurrence and regional adenopathy for head and neck cutaneous squamous cell carcinoma. Otolaryngol Head Neck Surg. 2017;156(5):863–9. 10.1177/0194599817697053 28322123

[27] Hinerman RW, Indelicato DJ, Amdur RJ, Morris CG, Werning JW, Vaysberg M, Kirwan J, Mendenhall WM (2008). Cutaneous squamous cell carcinoma metastatic to parotid-area lymph nodes. Laryngoscope..

[28] Hirshoren N, et al. Prognostic markers in metastatic cutaneous squamous cell carcinoma of the head and neck. Head Neck. 2017;39(4):772–8. 10.1002/hed.24683 28199044

[29] Jambusaria-Pahlajani A, Kanetsky PA, Karia PS, Hwang WT, Gelfand JM, Whalen FM, Elenitsas R, Xu X, Schmults CD (2013). Evaluation of AJCC tumor staging for cutaneous squamous cell carcinoma and a proposed alternative tumor staging system. JAMA Dermatol.

[30] Kelder W, Ebrahimi A, Forest VI, Gao K, Murali R, Clark JR (2012). Cutaneous head and neck squamous cell carcinoma with regional metastases: the prognostic importance of soft tissue metastases and extranodal spread. Ann Surg Oncol.

[31] Khandelwal AR, et al. Biomarker and pathologic predictors of cutaneous squamous cell carcinoma aggressiveness. Otolaryngol Head Neck Surg. (2016);155(2):281–8. 10.1177/0194599816641913 27095050

[32] Khurana VG, Mentis DH, O'Brien CJ, Hurst TL, Stevens GN, Packham NA (1995). Parotid and neck metastases from cutaneous squamous cell carcinoma of the head and neck. Am J Surg.

[33] Kreppel M, Krakowezki A, Kreppel B, Drebber U, Wedemeyer I, Mauch C, Zöller JE, Scheer M (2013). Podoplanin expression in cutaneous head and neck squamous cell carcinoma--prognostic value and clinicopathologic implications. J Surg Oncol.

[34] Kyrgidis A, Tzellos TG, Kechagias N, Patrikidou A, Xirou P, Kitikidou K, Bourlidou E, Vahtsevanos K, Antoniades K (2010). Cutaneous squamous cell carcinoma (SCC) of the head and neck: risk factors of overall and recurrence-free survival. Eur J Cancer.

[35] Makki FM, Mendez AI, Taylor SM, Trites J, Bullock M, Flowerdew G (2013). Prognostic factors for metastatic cutaneous squamous cell carcinoma of the parotid. J Otolaryngol Head Neck Surg.

[36] McLean T, Brunner M, Ebrahimi A, Gao K, Ch'ng S, Veness MJ, Clark JR (2013). Concurrent primary and metastatic cutaneous head and neck squamous cell carcinoma: analysis of prognostic factors. Head Neck.

[37] Mizrachi A, Hadar T, Rabinovics N, Shpitzer T, Guttman D, Feinmesser R, Bachar G (2013). Prognostic significance of nodal ratio in cutaneous squamous cell carcinoma of the head and neck. Eur Arch Otorhinolaryngol.

[38] Moore BA, Weber RS, Prieto V, El-Naggar A, Holsinger FC, Zhou X (2005). Lymph node metastases from cutaneous squamous cell carcinoma of the head and neck. Laryngoscope..

[39] Oddone N, Morgan GJ, Palme CE, Perera L, Shannon J, Wong E, Gebski V, Veness MJ (2009). Metastatic cutaneous squamous cell carcinoma of the head and neck: the immunosuppression, treatment, Extranodal spread, and margin status (ITEM) prognostic score to predict outcome and the need to improve survival. Cancer..

[40] Palme CE, O'Brien CJ, Veness MJ, McNeil EB, Bron LP, Morgan GJ (2003). Extent of parotid disease influences outcome in patients with metastatic cutaneous squamous cell carcinoma. Arch Otolaryngol Head Neck Surg.

[41] Peat B, Insull P, Ayers R (2012). Risk stratification for metastasis from cutaneous squamous cell carcinoma of the head and neck. ANZ J Surg.

[42] Pramana A, Browne L, Graham PH (2012). Metastatic cutaneous squamous cell carcinoma to parotid nodes: the role of bolus with adjuvant radiotherapy. J Med Imaging Radiat Oncol.

[43] Schmidt C, et al. Outcomes of nodal metastatic cutaneous squamous cell carcinoma of the head and neck treated in a regional center. Head neck. (2015);37(12):1808–15. 10.1002/hed.23843 24995842

[44] Sweeny, Larissa, et al. "Head and neck cutaneous squamous cell carcinoma requiring parotidectomy: prognostic indicators and treatment selection." Otolaryngology--Head and Neck Surgery 150.4 (2014): 610-617. 10.1177/0194599814520686 24474713

[45] Dyall-Smith D, et al. Cutaneous squamous cell carcinomas and papillomaviruses in renal transplant recipients: a clinical and molecular biological study. J Dermatol Sci. 1991;2(3):139–46. 10.1016/0923-1811(91)90059-7 1652277

[46] Vasan K, et al. Lymph node ratio as a prognostic factor in metastatic cutaneous head and neck squamous cell carcinoma. Head neck. 2018;40(5):993–9. 10.1002/hed.25066 29360276

[47] Tseros EA, et al. Prognostic significance of lymph node ratio in metastatic cutaneous squamous cell carcinoma of the head and neck. Ann Surg Oncol. 2016;23(5):1693–8. 10.1245/s10434-015-5070-6 26786095

[48] Wang JT, Palme CE, Morgan GJ, Gebski V, Wang AY, Veness MJ (2012). Predictors of outcome in patients with metastatic cutaneous head and neck squamous cell carcinoma involving cervical lymph nodes: improved survival with the addition of adjuvant radiotherapy. Head Neck.

[49] Moloney FJ, Comber H, O'Lorcain P, O'Kelly P, Conlon PJ, Murphy GM (2006). A population-based study of skin cancer incidence and prevalence in renal transplant recipients. Br J Dermatol.

[50] Ross AS, Schmults CD (2006). Sentinel lymph node biopsy in cutaneous squamous cell carcinoma: a systematic review of the English literature. Dermatol Surg.

[51] Karia PS, Jambusaria-Pahlajani A, Harrington DP, Murphy GF, Qureshi AA, Schmults CD (2014). Evaluation of American joint committee on Cancer, International Union against Cancer, and Brigham and Women's Hospital tumor staging for cutaneous squamous cell carcinoma. J Clin Oncol.

[52] Morand GB, Ikenberg K, Vital DG, Cardona I, Moch H, Stoeckli SJ, Huber GF (2019). Preoperative assessment of CD44-mediated depth of invasion as predictor of occult metastases in early oral squamous cell carcinoma. Head Neck.

