# Supplementary material for: Prognostic factors of head and neck cutaneous squamous cell carcinoma: a systematic review
Source: J Otolaryngol Head Neck Surg. 2021 Sep 7;50:54. doi: 10.1186/s40463-021-00529-7 (PMC8425113; doi:10.1186/s40463-021-00529-7)

**Additional file 1**

PubMed search strategy

(Prognosis/Broad[filter]) AND “predictor”[All Fields] OR predictors[All Fields] OR “Epidemiology”[MeSH Terms] OR “epidemiology”[All Fields] OR “Prospective studies”[MeSH Terms] OR “retrospective studies”[MeSH Terms]) AND (((((("carcinoma, squamous cell"[MeSH Terms]) AND "skin neoplasms"[MeSH Terms]) AND ("Head and Neck Neoplasms"[MeSH Terms]))) OR "cutaneous squamous cell carcinoma") OR "skin squamous cell carcinoma"))


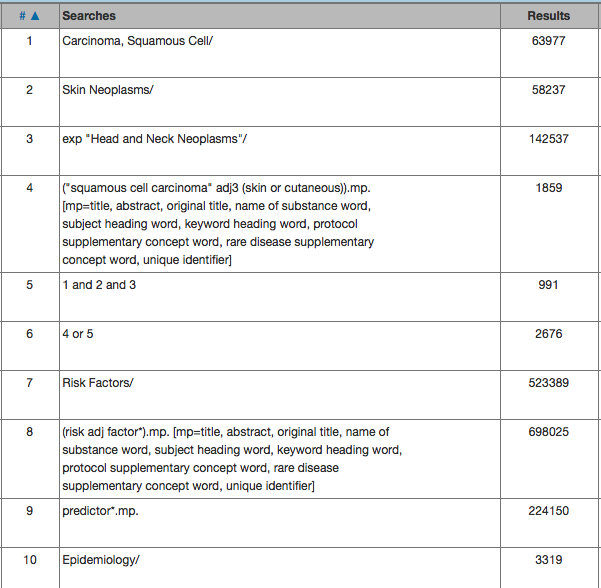
Medline search strategy


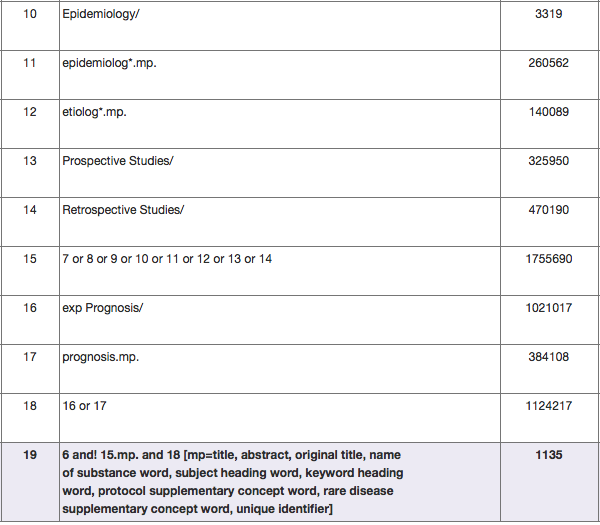

Supplement: Supplementary file 1 — Additional file 1. [file 40463_2021_529_MOESM1_ESM.docx]
